# Scaling up One Health: A network analysis in Lao PDR

**DOI:** 10.1016/j.onehlt.2023.100661

**Published:** 2023-12-12

**Authors:** Andrew Larkins, Soulasack Vannamahaxay, Vannaphone Puttana, Malavanh Chittavong, Fongsamouth Southammavong, Mayfong Mayxay, Davina Boyd, Mieghan Bruce, Amanda Ash

**Affiliations:** a School of Medical, Molecular and Forensic Sciences, Murdoch University, Perth, Australia; b Centre for Biosecurity and One Health, Harry Butler Institute, Murdoch University, Perth, Australia; c Faculty of Agriculture, National University of Laos, Vientiane, Lao Democratic People’s Republic; d Institute of Research and Education Development, University of Health Sciences, Ministry of Health, Vientiane, Lao Democratic People’s Republic; e Lao-Oxford-Mahosot Hospital-Wellcome Trust Research Unit, Mahosot Hospital, Vientiane, Lao Democratic People’s Republic; f Centre for Tropical Medicine and Global Health, Nuffield Department of Medicine, University of Oxford, Oxford, United Kingdom; g Lao One Health University Network, Vientiane, Lao Democratic People’s Republic; h Centre for Sustainable Farming Systems, Food Futures Institute, Murdoch University, Perth, Australia; i School of Veterinary Medicine, Murdoch University, Perth, Australia

**Keywords:** Collaboration, Mixed methods, Social network analysis, Organisational network analysis, Global Health

## Abstract

**Background:**

One Health focuses on sustainable health for humans, animals, and ecosystems. The approach has been well demonstrated, yet most efforts have not been scaled up. Understanding the organisations involved in scaling up processes is critical to translating research into practice. The Lao People's Democratic Republic has successfully implemented One Health projects for multiple decades; however, the organisational network has not been described and scaling up efforts have been limited.

**Methods:**

Data from organisations involved in One Health projects over the past five years were collected by key-informant interview or workshop. The network was investigated using a mixture of quantitative network analysis and qualitative thematic analysis.

**Results:**

The organisational network was quantitatively described as sparse and centralised. Organisations were required to harness pre-existing relationships to maximise scarce resources and make co-ordination and alignment of priorities more efficient. A lack of international organisations in the top 10% of resource sharing metrics suggests a potential disconnect between donors. This was reflected in the challenges faced by national organisations and a feeling of being stretched thin over numerous externally funded projects with donor-driven priorities.

**Conclusions:**

It appears that high-level political support for country ownership of development and aid priorities remains unrealised. Developing network capacity and capability may assist scaling up efforts and build resilience in the network and its core organisations. This may allow for the inclusion of more development, education, environment, and water, sanitation, and hygiene organisations that were perceived to be lacking. Future One Health programmes should focus on practical activities that do not overload staff capacity. There is much for One Health to learn about the art of scaling up and organisations are encouraged to include implementation science in their research to inform future scaling up efforts.

## Highlights

• One Health organisational network in Lao PDR is sparse and centralised.
• Network structure driven by relationships, resources, co-ordination, and priorities.
• Network capacity stretched by time-limited externally funded projects.
• Few development, education, environment, and WaSH organisations are present.
• New projects should consider capacity, feasibility, and implementation research.

## 1 Introduction

The One Health philosophy has been present in society for centuries, if not millennia [1]. Its application relies on multiple sectors working together to sustainably enhance the health of humans, animals, and the environment [2]. The recent COVID-19 pandemic has rekindled interest and funding of the approach, although previous waves of support have ultimately been limited in their ability to sustainably improve health on a large scale [[3], [4], [5], [6], [7], [8], [9]]. There are many challenges to operationalising One Health, ranging from broad issues of global governance to the specific nature of individuals and their relationships [[10], [11], [12]]. These challenges should not stop the application of One Health approaches; however, they must be taken into consideration.

One Health is not alone in the challenges it faces when trying to translate research into practice. Scaling up interventions and innovations has been a focus of the human health sector in recent decades, yet there is still a significant gap between what is known and what is done, particularly in low resource settings [[13], [14], [15]]. A key aspect of scaling up is understanding the individuals and organisations that may promote, support, adopt, or implement research [15]. Network analysis provides an opportunity to identify and understand these organisations and aide scaling up efforts [16,17].

One Health and the closely related Ecohealth approaches have been applied in the Lao People's Democratic Republic (PDR) for multiple decades [8]. This has included successful discrete projects on pandemic threats, zoonotic pathogens, antimicrobial resistance and more [[18], [19], [20], [21], [22], [23]]. These successes have involved a network of organisations and relationships whose structure and experiences have yet to be investigated and learnt from. One Health networks have been the subject of extensive research in recent years [24,25] though few studies have focussed on the experience of operationalising One Health in southeast Asia [23,[26], [27], [28]]. This paper aims to describe the One Health organisational network in Lao PDR and investigate why the network is structured the way it is and what changes might strengthen or help to scale up One Health approaches in the future.

## 2 Materials and methods

### 2.1 Design and data collection

A mixed methods convergent design was applied to investigate the One Health organisational network in Lao PDR. Organisations were considered at their highest level and departments were not considered as separate organisations. A semi-structured interview protocol was developed and adapted for workshop use (Appendix A). A one-day workshop and independent one-hour interviews were used to collect data on the organisational network and participant experiences in One Health projects (AL, FS, MC, SV, and VP). During the workshop, participants were divided into groups of six to eight people and completed the interview protocol as groups. Each group presented their answers to the workshop with further discussion facilitated between groups.

To collect quantitative organisational data, a list of study organisations was created by the authors and their professional network. This list was cross-checked against informal searches of PubMed and Google using the terms “Lao One Health” or equivalent. The organisational list was snowballed during data collection with each participant reviewing the list and adding missing organisations. This iterative creation of the sampling frame was implemented to reduce selection and recall bias [29].

During the interviews, participants reviewed the list of study organisations and completed a network table to identify organisations that they shared information or resources with during One Health projects in the past five years. Each organisational relationship was scored for information sharing (0: no sharing – 5: more than three times a year) and resource sharing (0: zero – 5: all resources) (Appendix A). During the workshop each group of six to eight participants reviewed the list of study organisations then created a network diagram by drawing the organisations that they had shared information or resources with. Each relationship was then scored using the same Likert scale as in the interviews. Diagrams were created by consensus within each group and were later transcribed into network tables.

Qualitative questions explored the successes, challenges, and scaling up of One Health projects. Interviews and the workshop followed the same semi-structured protocol (Appendix A). During the workshop, participants discussed their responses within their groups before presenting their findings to the wider room for further facilitated discussion. Audio recordings of interviews were transcribed verbatim, while workshop discussions were summarised based on written notes, participant follow-up, and facilitator recall (AL and SV).

Lao organisations participated in the workshop, whilst international organisations and some Lao organisations provided data through semi-structured interviews. In total 45 individuals from 23 organisations participated in the study. Twelve individuals from 8 organisations provided data in key informant interviews and 33 individuals from 14 organisations participated in the workshop (Appendix B). The purpose of using two separate data collection methods was to separate donors and recipients to reduce sponsor bias. Written consent was provided by all participants prior to data collection. Ethical approval was granted by the Lao National Ethical Committee for Health Research (59/NECHR) and the Murdoch University Human Research Ethics Committee (2022/057).

### 2.2 Data analysis

Study organisations were categorised by their location, primary focus and type based on participant responses. Network tables were transcribed into a single undirected network matrix for network analysis using Gephi [30]. Where organisational relationships had been scored more than once, the mean score was applied. Degree, weighted degree, betweenness centrality, and closeness centrality were used to assess the influence of each organisation in the network. Density, average degree, average distance, and diameter were calculated to describe the entire network (Table 1) [[29], [30], [31]]. Qualitative data were analysed using mixed inductive-deductive thematic analysis in NVivo [32]. Data were coded using predetermined themes of successes, challenges, and scaling up before being developed into sub-themes during analysis. Common sub-themes were summarised as the key considerations for the One Health network.

**Table 1:** A brief description of network analysis metrics assessed for information and resource sharing during One Health projects in Lao PDR.

Perspective	Metric	Description
Organisation	Degree	The number of connections an organisation has.
	Weighted degree	The sum of an organisation's connections multiplied by their respective strength.
	Betweenness centrality	How often an organisation lies between two others.
	Closeness centrality	How close an organisation is to all others.
Network	Density	Proportion of relationships present in the network.
	Average degree	Average number of connections for an organisation.
	Average weighted degree	Average number of connections for an organisation multiplied by the average strength of connections.
	Average distance	Average number of relationships that separate two organisations.
	Diameter	The number of relationships between the two furthest organisations in the network.

## 3 Results

### 3.1 Quantitative network analysis

**Fig. 1:**
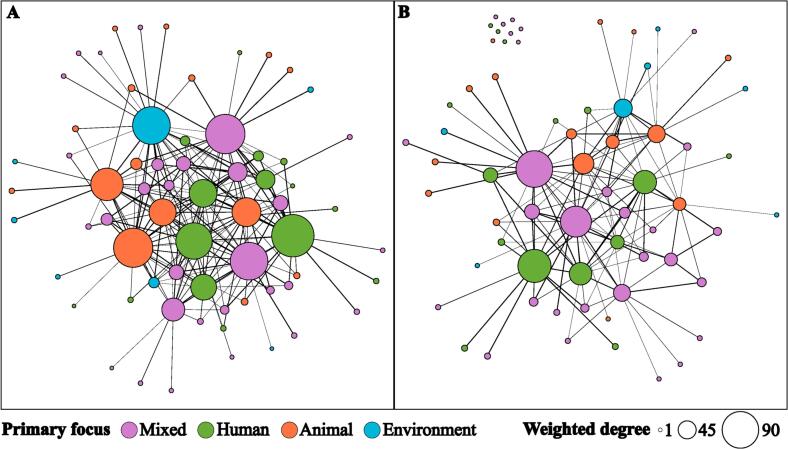
Network diagrams of organisations sharing information (A) and resources (B) on One Health projects in Lao PDR. Organisation colour represents primary focus. Size relative to the weighted number of connections (weighted degree).

**Fig. 2:**
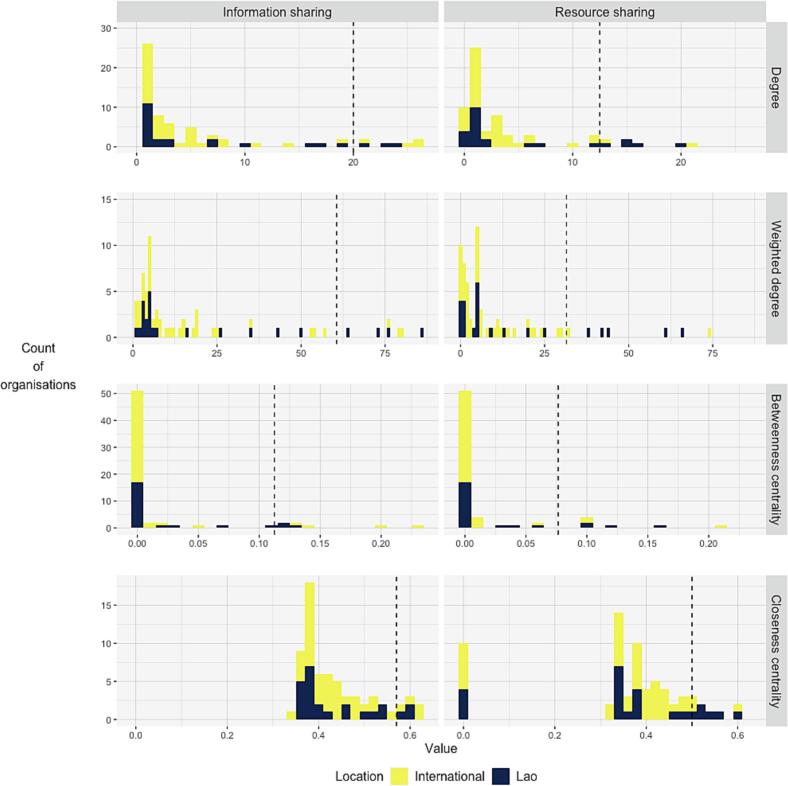
Histograms of key organisation metrics for information (left) and resource sharing (right). Degree (top) is the number of connections for an organisation. Weighted degree (second from top) is the number of connections multiplied by the respective strength of each connection with an organisation. Betweenness centrality (third from top) is a measure of how often an organisation lies between two others. Closeness centrality (bottom) is a measure of how close an organisation is to all others. Dashed line represents 90th percentile. Complete data available in Appendix B.

Sixty-six organisations were identified in the network, with 202 information sharing relationships and 194 resource sharing relationships present. Most organisations were classified as international, had mixed focus, and were research organisations (Table 2; Appendix B). The sizeable proportion of organisations with mixed focus was due to the broad remit of large organisations, such as universities and some donor organisations. The overall network density was low with only 9% of information sharing and 6% of resource sharing relationships realised (Table 3). Despite this, the diameter of the network was small, and the most removed organisation could connect with an organisation on the other side of the network by harnessing four relationships (Table 3). The average organisation in the network shared information with six others; however, only shared resources with four of these organisations (Table 3). On average, two organisations were separated by two or three relationships (Table 3). These network-level measures describing an average organisation are somewhat misleading due highly skewed nature of the organisation-level metrics. Examining the organisation metrics, there are a small number of organisations that are heavily involved in the network with many connections and a large number with few connections (Fig. 1; Fig. 2; Appendix B). Eleven organisations formed the top 10% of all organisation metrics. Seven of these were included in the top 10% for both information and resource sharing (Fig. 2; Appendix B). These leading organisations were relatively evenly split by type; yet, there was only one organisation with a primary focus on the environment (Appendix B).

**Table 2:** A summary of organisations involved in One Health projects in Lao PDR categorised by location, primary focus and type. Complete data available in Appendix B.

Category	Count (%)
**Location**	
International	42 (64%)
Lao PDR	24 (36%)
**Primary focus**	
Mixed	29 (44%)
Animal	15 (23%)
Human	15 (23%)
Environment	7 (11%)
**Organisation type**	
Research organisation	23 (35%)
Government organisation	19 (29%)
Non-government organisation	15 (23%)
Multilateral organisation	9 (14%)
**Total**	**66 (100%)**

**Table 3:** Key network metrics for organisations sharing information and resources during One Health projects in Lao PDR.

Network metric	Information sharing	Resource sharing
Density	0.09	0.06
Average degree	6.10	3.91
Average weighted degree	19.12	11.57
Average distance	2.36	2.52
Diameter	4	4

### 3.2 Key considerations for One Health projects

The experiences of network participants suggest four key considerations that may explain the network structure. Relationships, resources, co-ordination, and priorities were able to describe why the network was centralised with a low density.

#### 3.2.1 Relationships

When considering successful One Health projects, it was consistently agreed that good relationships were essential. However, it was believed that the requirement to invest and maintain relationships meant that organisations tended to only share information and resources with those closest to them. As explained by a senior staff member from a Lao research organisation:

*“If you have good connections with others then you get lots of support and it's easier to collaborate if you already have a connection with them. That's makes the approach successful. Mostly those we bring in, even though they are in different departments, institutions, or domains, we already know them. It's better that way and easier to develop something together. We bring them in early, starting from design. If we involve them early, then when you want to implement it's much easier”* [Research organisation (RO) 6].

Personal connections were believed to be critical and were often stated as the primary point of entry for any work. As described by one participant, *“interpersonal relationships are more effective than international relationships, as you see local resource mobilisation. If the provincial governor promises a project, then he keeps the promise”* [Multilateral organisation (MLO) 4]. It was commonly found that poor relationships may derail projects, with another participant experiencing that *“personal interests, either expressed or not expressed, can really block the discussion, as everyone wants to keep their small label that I'm the lead in this area. It's very difficult”* [Non-government organisation (NGO) 1].

Some participants found building new relationships challenging due to the time and effort required. One government staff member felt that *“if you're really collaborating with other institutions, you can't be everywhere”* [Government organisation (GO) 15]. Others suggested that the term One Health lacks clarity and is unknown to some stakeholders, particularly those from cross-cutting sectors.

*“The real comprehension of the word* (One Health) *is difficult for people. The key is that people properly understand One Health and why we need it*. *We need to increase advocacy and promotion, otherwise it will be what we have faced before”* [MLO 2].

*“Lots of people want to promote One Health, but lots of researchers make it something very complicated. Really you can do One Health very simply. It's not an expertise, it's a way of doing things. We need to simplify this concept for everybody so that it's accessible to everybody”* [NGO 1].

#### 3.2.2 Resources

As expected in a low-resource setting, resources were a frequently discussed topic. A common sub-theme was that the scarcity of resources considerably impacted the ability of organisations to collaborate. It was frequently revealed that organisations tend to focus on sharing available resources with established partners. One participant described that available resources may quickly become diluted may cause conflict in projects where there are insufficient opportunities for contribution or recognition:

“*A small budget is shared. Then there's the responsibility. The ownership for the activities becomes unclear. Who will take lead is unclear and then there is no lead. No one wants to be second”* [RO 6].

Limited capacity and capability of human resources was another consistent sub-theme, with skilled national staff reported to be overloaded and *“pulled around different projects”* [GO 1]. In terms of capability, one workshop participant surprisingly revealed that this study's workshop was, *“the first time that anyone has really explained to me what One Health means, and I've been working on One Health projects for a long time”* [GO 2]. This experience was reiterated during an interview with a multilateral organisation, who stated that the limited availability of capable technical staff makes operationalising One Health a challenge, as *“every time you organise a workshop it's new people. The other ones aren't available, and you have to explain everything again”* [MLO 4].

Multiple organisations commented that there were few highly skilled One Health practitioners available. It was reported that international organisations apply a train-the-trainer approach to build staff capacity and capability; however, MLO staff member was sceptical of the method due to the external nature of their funding. They believed that *“in the end it's not very sustainable. As soon as we leave, the next people entering the department don't get trained up* (as there is no funding for the trainers to conduct any training)*”* [MLO 2]. There was a strong consensus amongst participants that external funding of projects was a considerable challenge. As stated by an international NGO staff member, *“the financial piece in Laos* (Lao PDR) *is hard. Unless you're rolling into the next thing, a lot of that work just gets dropped”* [NGO 3]. The short-term nature of externally funded projects was described by many as limiting monitoring, evaluation, and learning efforts. One participant reported that this substantially impacted on the long-term sustainability of activities:

*“Most of the projects having funding for one or two years. We need time to engage with the communities. We do the training first, we do the investments, and then we stop. We train them on technical issues and tell them the content of what they should be doing but they don't have any training as facilitators. To be effective they need that. We need to be able to follow them and support them before it becomes sustainable”* [NGO 1]*.*

A third sub-theme commonly reported was that integrating One Health into routine work was key to successful approaches. Participants believed that activities should be *“based on budget, staff that have the ability, and connecting and integrating work between sectors”* [GO 1]. One participant suggested that:

*“If we can find ways to build One Health into regular work and make it a responsibility of people who are receiving a salary it will help with capacity and sustainability. Sometimes you have to start small. It can't be this pie in the sky thing that gets dropped here. You have to start at the most practical and work your way towards the gold standard”* [NGO 3].

A government staff member had found this approach had previously resulted in *“a strong nucleus* (of experienced and skilled staff) *to show others how to do the work, but you must find the right people”* [GO 15].

#### 3.2.3 Co-ordination

It was a regular discussion that even when sufficient resources were available, the co-ordination of a One Health approach presents *“a big challenge”* [RO 6]. To manage co-ordination, participants agreed that organisations try to engage with existing partners early in the project process.

“*You need to have people from different backgrounds and organisations. It's a lot of people that need to be available every time. There are so many different organisations*. *To make one point or get a consensus is hard. Sometimes we have three meetings and can't get a conclusion. That makes the One Health approach difficult. We must discuss a lot, but action is little sometimes”* [RO 6].

Participants suggested that the lack of resources and co-ordination meant that in some cases official meetings have fallen away, existing in title only. One senior participant confirmed *“there is a list of names. My name is on that list, but I've never been to a meeting”* [MLO 2]. Some organisations reported that co-ordination was challenged by the lack of a formal network.

*“Keeping abreast of what is happening is challenging. I don't know the solution, but I think these projects would be more efficient and we'd find better ways to move forward in terms of sustainability if we could keep on top of what's happening”* [NGO 3]*.*

All participants strongly supported the resurrection of an active national One Health taskforce or committee and, despite the challenges of co-ordination, most believed that scaling up of the One Health network was possible. As summarised by one participant, *“it wouldn't be that expensive, but someone needs to take ownership of it”* [MLO 2].

#### 3.2.4 Priorities

The use of existing policies, memorandums of understanding, or other agreements was identified as a key to success. It was reported that such agreements allowed for roles, responsibilities, and contributions to be clearly defined prior to implementation and for partnering organisations to have agreed objectives and understanding of projects. For example, for avian influenza there is “*a memorandum of understanding* (which outlines that) *doctors do humans, vets do animals, but there is good communication and then clear responsibilities in the field”* [GO 1].

There was agreement that “*it's important to have strong relationships with government”* due to the social and political structure of Lao PDR, and that projects have generally been more successful when they *“have positive relationships with government partners and are not trying to do things on their own”* [NGO 3]. Whilst participants were unanimous that government support is critical, it was also frequently noted that funding landscape has made sustained progress difficult. Currently, *“financial support comes from outside, mainly donors or development partners. Each funder has their own target, each player has its own target, it's always a balancing act”* [MLO 4]. A common discussion during the interviews and workshop was that many externally funded One Health projects may not be consistent with national priorities.

*“Projects get proposed and picked up because there are external funds available. They aren't necessarily something the government views as valuable in the long term. Projects need to fit the national priorities. They need to be useful”* [NGO 3].

Participants believed that there are many opportunities to implement One Health approaches in Lao PDR; however, agreed that, “*budget is key and donors change priorities and staff, making it difficult”* [RO 9]. There was a strong call across interviews and the workshop for a focus on practical and feasible actions.

*“There's a lot we could discuss* (besides avian influenza and antimicrobial resistance) *but that's what gets paid for by foreigners or external funds. We need to focus on practical things to work on together, rather than meeting with the same people and talking about influenza every time but it's the fashion and availability of the budget”* [MLO 2].

A clear sub-theme that emerged from analysis was that finding tangible actions to collaborate on with cross-cutting sectors such as development, education, and WaSH is highly relevant to scaling up One Health in Lao PDR. As poignantly concluded in one interview:

*“What are you going to do with the money? In a country like Laos* (Lao PDR) *where much of the rural population don't have a toilet or running water. Maybe if they want to do something on One Health then maybe they should be looking more closely at sanitation and poverty”* [MLO 2].

## 4 Discussion

The mixed methods applied in this study have demonstrated a sparse and centralised network operating in an environment largely controlled by external funding. Given the limited resources and demands of co-ordinating multiple disciplines, organisations tend to harness existing relationships rather than investing in untested partners. Consequently, organisations at the core of the network have long-standing relationships that they rely on to conduct most of the One Health activities in Lao PDR. This centralised network may be a double-edged sword, particularly given its low density. If one of the core organisations were to have its capacity or capability reduced it would markedly affect One Health operations. Developing the density of the network may increase capacity, assist scaling up efforts, and create resilience that protects the network from potential shocks and changes.

Given the network structure and preference for existing and formal arrangements, it seems reasonable that one of the core organisations could take ownership and facilitate co-ordination of the wider network through an agreed mechanism. Due to the small diameter of the network such efforts may offer substantial reward. The Lao One Health University Network already operates in this role for the academic sector and could possibly be expanded to include other types of organisations. Such an approach may have the added benefit of exposing early- and mid-career professionals to One Health training opportunities. Given the skills gap currently described in the network, activities focussed on reducing the gap would significantly strengthen One Health in Lao PDR.

A focus on workforce planning and practical activities may provide possible avenues that support network capacity, capability, and co-ordination. Such efforts previously, have led to ongoing collaborative approaches for avian influenza and other zoonotic diseases [18,23]. The experience of staff overload and call for feasible activities described in this study should be heeded. Many projects currently appear to provide little detail on how existing human capacity will be managed and we appeal for more thorough reviews of feasibility and capacity before overwhelming existing workforces. There are various tools developed by multilateral organisations that can provide useful frameworks for assessing One Health capacity prior to project implementation [33], however more specific details will often be required depending on the programme. Similarly, One Health projects in Lao PDR often appear to be missing the implementation science and research required for scaling up. Projects often perish without providing any form of guidance that countries or future projects can engage with in a tangible manner. When guidance is provided it needs to be grounded in feasibility so that it may play a role in informing workforce planning. The art of scaling up is yet to be realised in One Health, as demonstrated by the very failure to scale up projects that have often been evaluated as cost-effective [[6], [7], [8], [9]].

We encourage organisations to include implementation research and scaling up in their proposals and encourage donors to provide recipients with the support required to complete such work, as is now being seen in public health programmes [34]. A focus on tangible objectives and developing the current One Health network with increased advocacy may allow for a wider range of organisations to become engaged. The lack of development, education, WaSH, and environmental organisations active in the Lao One Health network has been reported in other networks and a sectoral divide is still present in academic literature [[24], [25], [26]]. If One Health is to be scaled up further in Lao PDR, organisations must form relationships and work with those in cross-cutting sectors. There is much to be learnt from the private sector where collaboration is common and often tied to success. Reaching beyond academia, public policy, and technical consultants is encouraged and supported by the case of scaling up the Avahan program for HIV control in India [35,36].

Developing the One Health network further will require increased and revised funding arrangements. The challenges of managing externally funded programmes have frequently been found in other One Health networks [25,37,38]. External funding of One Health has now reached a remarkable rate in some places, with greater than 90% of initiatives in Africa being externally funded [38]. Without improved alignment, the memory and development of national organisations and staff will remain challenged as they are required to constantly pivot to address various donor priorities. It is no surprise that even in well-resourced projects, national organisations may lack motivation if they received limited ownership [39]. These experiences are not novel or constrained to the realm of One Health and there have been many high-level political declarations calling for country ownership of development priorities [[40], [41], [42]]. Nevertheless, this study suggests that these affirmations have not yet reached their aspirations and increasing funding alignment towards national priorities may assist in developing the One Health network in Lao PDR.

In terms of study limitations, the semi-quantitative and qualitative nature of this study makes it susceptible to various forms of bias. Only the perceived network has been described and some organisations will remain hidden because they were not perceived to participate. In addition, successful and unsuccessful projects and relationships were not formally defined and this was left to participants' own value judgements. One future solution to this limitation could be to examine formal funding arrangements and project evaluations; however, such data may be difficult to obtain. Attempts have been made to reduce the level of selection and recall bias through a snowball sampling method and the application of multiple data collection tools [29]. Only one quarter of organisations in the network were sampled, yet those sampled were the most frequently identified in the network and represent considerable network coverage. All organisations with greater than five and ten resource and information sharing relationships respectively were sampled. Given the lack of previous One Health studies applying quantitative network analysis, contextualising the sample size and metric results is challenging.

Future work should strive for increased network coverage and the inclusion of more outlying organisations may provide different perspectives on collaboration. A comprehensive network analysis may also include different sections of large organisations, for example different departments within universities or provincial and district departments within ministries. Further breakdown of organisations is likely to reveal heterogeneity within large organisations and may identify areas for rapid improvement. This study was primarily descriptive and exploratory in nature. Detailed evaluations of One Health projects and programmes should be used to suggest where specific network improvements may be made [[43], [44], [45]]. Such efforts should focus on how organisations can create and harness the formal and informal relationships that were believed to be critical to success. The inclusion of quantitative network analysis in these evaluations may provide an additional measure on which to compare and evaluate different One Health programmes. Finally, knowledge, attitudes, and practices were not investigated in this study; however, they may impact the structure of the network and should be investigated in the future. Some organisations will have been missed because they were not active during the recall period. This reflects the dynamic nature of networks, and the authors are aware of new projects that have not yet commenced. Given the COVID-19 pandemic, it is likely that there will be an influx of One Health projects in coming years that will see this dynamic network evolve. Network analysis has the potential to monitor the evolution of the One Health network over time.

## 5 Conclusions

Despite a resource constrained environment, the organisational network in Lao PDR has been able to provide successful examples of One Health projects [8,[18], [19], [20], [21], [22]]. Given the structure of the network this highlights the importance of those organisations at the core of the network. Much of the success has been due to the resolve and capability of individual staff and their ability to build working relationships with those closest to them. These experiences and relationships provide optimism that One Health can be scaled up in Lao PDR if the network is developed with a focus on relationships, resources, co-ordination, and priorities. It is hoped that this descriptive study can inform the design of future One Health evaluations that investigate how the network and future projects can be tailored for success.

## Author contributions

AL, MB, MC, FS and AA conceived study. All authors were involved in study design. AL, FS, MC, SV and VP collected data. AL and SV analysed the data. AL drafted the original manuscript. All authors reviewed and approved draft and final manuscripts.

## Funding

This research was funded by the 10.13039/501100000974Australian Centre for International Agricultural Research (LS/2014/055). AL's contribution was also funded by a Murdoch University Strategic Scholarship. The funders had no role in study design, data collection and analysis, decision to publish, or preparation of the manuscript.

## Declaration of Competing Interest

The authors declare that they have no competing interests.

## Data Availability

Original data are available from the corresponding author upon reasonable request.

## Appendix A Supplementary data

Supplementary material 1 Appendix A. Data collection protocols. Image 1

Supplementary material 2 Appendix B. Organisation metric results. Image 2
