# Supplementary material for: Scaling up One Health: A network analysis in Lao PDR
Source: One Health. 2023 Dec 12;18:100661. doi: 10.1016/j.onehlt.2023.100661 (PMC10761780; doi:10.1016/j.onehlt.2023.100661)
Supplement: Supplementary file 1 — Supplementary material 1 Appendix A. Data collection protocols. [file mmc1.docx]

**KEY INFORMANT INTERVIEW PROTOCOL**

1. **INTRODUCTION**
2. **Background**

*Introduce researcher(s) and thank participant for time.*

*Read through information letter and provide participant with a copy.*

1. Do you have any general questions about the project or this study?
2. **This interview**

This interview is semi-structured, and the questions relate to the work that your organisation does around One Health.

Before we begin, I would like to provide you with some more information about this study and make sure that you are happy to participate and understand what is involved.

1. **Consent**

*Read through consent form and obtain written consent from participant.*

1. Do you have any questions before we begin?

Thank you again for agreeing to participate. I will start the recording *(if audio recording consent obtained)*.

1. **Interview Details**

| Date |  | Time |  |
| --- | --- | --- | --- |
| Location |  | Enumerator |  |

**2. BACKGROUND AND ORGANISATIONAL PRIORITIES**

1. **How long have you worked on One Health projects?**

| Less than 5 years | 5 – 10 years | More than 10 years |
| --- | --- | --- |

1. **One Health is focussed on human, animal, and environmental health. Which of these core areas is the primary focus for your organisation?**

| Human health | Animal health | Environmental health |
| --- | --- | --- |

1. **What kind of One Health issues does your organisation currently work on?**

| Anti-microbial resistance (AMR) | Endemic zoonotic diseases |
| --- | --- |
| New and emerging diseases | Other (please specify below) |
|  | |

1. **What sorts of activities is your organisation currently involved with?**

| Policy | Disease surveillance |
| --- | --- |
| Research | Disease control |
| Other (please specify below) |  |
|  | |

**4. STAKEHOLDER NETWORK**

**We will now move onto looking at the organisations that you work with and try and describe your relationship with them. This will allow us to use network analysis to examine the One Health network in Laos.**

***Provide participant with a copy of network table.***

**The first column lists the organisations that may be involved in One Health projects. We have found them by searching the literature, speaking with contacts, and interviews.**

1. **Please mark any organisation that you work with.**
2. **Are there any organisations missing that should be included?**

***Add the name and contact details of new organisations to bottom of network table***

**If you think of any throughout the interview, please let me know. The more organisations that we can speak with, the more useful the results will be.**

**5. INFORMATION AND RESOURCE SHARING**

**We are considering two aspects of how different organisations work together on One Health projects: information sharing and resource sharing.**

**Sharing information may be in the form of meetings, workshops, emails, phone calls, etc.**

**Sharing resources can apply to funding, staff, buildings, laboratory equipment, vehicles, etc.**

**We will score each of these on a scale of 0-5.**

**We will now try to fill in this table thinking about projects that your organisation has been involved in over the past 5 years.**

***Column 2***

1. **How often does your organisation share information on One Health projects with each of the organisations?**

***Column 2***

1. **What scale of resources does your organisation share with each of the organisations on One Health projects?**

**Feel free to keep your copy and discuss it with anyone else in your organisation that works on One Health. You can always send me an updated copy or any additional thoughts that you might have.**

| **ORGANISATION** | **INFORMATION SHARING (0-5)** | **RESOURCE  SHARING (0-5)** |
| --- | --- | --- |
|  |  |  |
|  |  |  |
|  |  |  |
|  |  |  |
|  |  |  |
|  |  |  |
|  |  |  |

**6. COLLABORATION**

1. **Thinking about the network of organisations that we’ve just discussed and some of the previous projects that you’ve been involved with, what has made for a good relationship between organisations?**
   1. **Example**
   2. **Why were these relationships it successful?**
   3. **What was been the outcome of some of these collaborations?**
   4. **What were the key factors for success?**

|  |
| --- |

1. **Why do you think some relationships between organisations fail to live up to their potential?**
   1. **Example**
   2. **Why were these relationships unsuccessful?**
   3. **What was learnt from these experiences?**
   4. **Have these experiences changed how things are done?**

|  |
| --- |

**7. SCALING UP ONE HEALTH**

1. **If One Health interventions are proven to be effective, economic, and acceptable, do you think they could be successfully scaled up to more villages? Why? Why not?**

|  |
| --- |

1. Do you think One Health interventions could be successfully scaled up as part of routine work?
   1. Why?
   2. Why not?

|  |
| --- |

1. Who are the most important stakeholders that need to be engaged to make this possible?
   1. Why these people?

|  |
| --- |

**8. SUMMARY**

**Thank you for your time and speaking with me today.**

**Is there anything else you think might be relevant to this study or the wider project?**

**Is there anyone you would recommend I speak with for this study?**

**Would you like to be updated on the progress of this study and the project?**

**If you would like to speak more my details are on the information sheet.**

**ONE HEALTH WORKSHOP PROTOCOL**

The workshop uses the same questions are interview protocol. However, a facilitator will present the questions and have small groups discuss and present their findings. A dedicated note-taker will be present on each table.

**Agenda**

| Time | Session |
| --- | --- |
| 8:30am | Official welcome |
| 9:00am | A history of One Health in Lao PDR |
| 9:30am | Background and organisational priorities |
| 10:00am | Break |
| 10:15am | Stakeholder network analysis |
| 12:00pm | Lunch and group photo |
| 1:00pm | One Health collaboration |
| 2:15pm | Break |
| 2:30pm | Scaling up One Health |
| 3:45pm | Summary of workshop and next steps |
| 4:00pm | Official close |
